# Association between Cesarean Section and Weight Status in Chinese Children and Adolescents: A National Survey

**DOI:** 10.3390/ijerph14121609

**Published:** 2017-12-20

**Authors:** Jingjing Liang, Zheqing Zhang, Wenhan Yang, Meixia Dai, Lizi Lin, Yajun Chen, Jun Ma, Jin Jing

**Affiliations:** 1 Department of Maternal and Child Health, School of Public Health, Sun Yat-Sen University, Guangzhou 510080, China; stliangjingjing@126.com (J.L.); yangwhan@mail.sysu.edu.cn (W.Y.); daimx@mail2.sysu.edu.cn (M.D.); linlizi@mail2.sysu.edu.cn (L.L.); chenyj68@mail.sysu.edu.cn (Y.C.); 2 Department of Nutrition and Food Hygiene, Guangdong Provincial Key Laboratory of Tropical Disease Research, School of Public Health, Southern Medical University, Guangzhou 510515, China; zzqaa501@163.com; 3 Institute of Child and Adolescent Health, School of Public Health, Peking University, Beijing 100871, China; majunt@bjmu.edu.cn

**Keywords:** cesarean section, overweight, obesity, child, adolescent

## Abstract

Previous research on the association between cesarean section (CS) and childhood obesity has yielded inconsistent findings. This study assessed the secular trend of CS and explored the relationship between CS and the risks of overweight and obesity in Chinese children and adolescents. Data came from a national multicenter school-based study conducted in seven provinces of China in 2013. Covariate data including weight, height and delivery mode were extracted. Poisson regression was applied to determine the risk ratios (RRs) and 95% confidence intervals (CIs) for the risks of overweight and obesity associated with the delivery mode. A total of 18,780 (41.2%) subjects were born by CS between 1997 and 2006. The rate of CS increased from 27.2% in 1997 to 54.1% in 2006. After adjusting for major confounders, the RRs (95% CI) of overweight and obesity among subjects born by CS were 1.21 (1.15 to 1.27) and 1.51 (1.42 to 1.61), respectively. Similar results were observed in different subgroups stratified by sex, age, and region. In summary, the CS rate increased sharply in China between 1997 and 2006. CS was associated with increased risks of overweight and obesity in offspring after accounting for major confounding factors.

## 1. Introduction

The past two decades have witnessed a dramatic increase in the rate of births by cesarean section (CS) all over the world [1,2]. In the United States, this rate increased from 20.6% in 1997 to 31.5% in 2009 [3] and the rates in European countries ranged from 19% to 33% between 2002 and 2009 [4]. In China, the CS rate increased from 2.0% during 1978 and 1985 to 36.6% during 2006 and 2010 and reached 54.9% in 2011 [5], nearly four times the World Health Organization recommended highest proportion of 15% [6]. A growing body of literature suggests that birth by CS predisposes offspring to adverse health outcomes in later life, including asthma [7], respiratory morbidity [8], type-1 diabetes mellitus [9], and allergies [10].

Childhood obesity is a global public health issue [11], and the effects of CS on childhood obesity have already been evaluated in many studies involving varied populations, such as Americans [12], Danish [13], Australian [14], Brazilian [15], and Canadian [16]. However, these studies have yielded inconsistent results. Three recent meta-analyses have examined whether CS increases the risk of later overweight and obesity. Two revealed 24–34% increases in the risk among children and adolescents delivered by CS [17,18]. In contrast, using bias-adjusted meta-analysis, Sutharsan et al. found that the observed effect of CS on overweight and obesity among offspring was largely attributable to residual confounding and publication bias, and thus no compelling evidence supported an association between CS and the risk of overweight and obesity [19]. Subsequent studies also reported inconsistent results. Some studies observed a positive relationship between the delivery mode and offspring weight status [20,21], whereas others found no association [22,23]. Therefore, it remains unclear whether the delivery mode independently predicts the occurrence of obesity in later life.

In addition, some studies have indicated that CS may increase the risk of obesity in early childhood, but not in later life [15,24]. This effect may be attributable to increased exposure to postnatal obesogenic environmental factors such as dietary habits and physical activity increases with older age, which may mask the association between CS and obesity in later life [25]. Few studies have investigated the relationship of CS with overweight/obesity in Chinese children, and most were local studies of children younger than 7 years [21,26,27]. Therefore, nationwide population-based studies are needed to address this issue. Our study aimed to estimate the overall CS rate and related secular trend in China from 1997 to 2006 and to examine whether CS correlated with childhood overweight and obesity after adjusting for potential confounders.

## 2. Materials and Methods

### 2.1. Study Population

Data were obtained from a baseline survey of a national multicenter school-based obesity intervention project conducted in seven provinces of China (“Development and Application of Student Critical Diseases Prevention and Control Technology and Related Standards”; Registration number: NCT02343588). The design and sampling procedures have been reported in detail elsewhere [28]. For our study, the valid weight and height measurements for 45,608 primary and middle school students between 7 and 16 years of age were recorded. Participants with missing or implausible data regarding the birth date, delivery mode, or sex were excluded. Participants with other missing data are indicated in Table 1. A total of 39,786 complete cases were included. All participants voluntarily joined this study, and their parents provided written informed consent. Approval for the study was obtained from the Ethical Committee of Peking University.

### 2.2. Anthropometric Data

Trained clinicians and nurses collected anthropometric data between September and November 2013. All children enrolled in the study underwent physical examinations to obtain their weights and heights according to standard guidelines. Height was measured to the nearest 0.1 cm. Weight was measured to the nearest 0.1 kg with the child wearing only underwear. Both anthropometric measurements were digitally measured twice for each subject, and the average was calculated. Intra-class correlation coefficients (ICCs) were used to assess the inter-observer reliability and test-retest reliability. The ICCs of height and weight were 0.993 and 1.0, respectively. Body mass index (BMI) was calculated as the ratio of weight (in kg) to height squared (in m^2^) and was converted to an age- and sex- specific BMI Z-scores [29]. We defined overweight (Z-score from 1 SD to <2 SD) and obesity (Z-score ≥ 2 SD) according to the World Health Organization’s Child Growth Standards [30]. 

### 2.3. Potential Covariates

Information regarding the mode of delivery, socioeconomic characteristics, and other potential early life risk factors for obesity were obtained using a standard self-reporting parental questionnaire. The mode of delivery was a binary variable, cesarean or vaginal. We classified parental education level into three categories: none/primary (<6 years of schooling), secondary (7–12 years), and university or higher (>12 years). Missing categories were created for variables with missing values.

Lifestyle factors were surveyed with a student questionnaire that included six items on dietary behaviors, four items on sedentary behaviors, and three items on physical activities. Children under 12 years were requested to complete the questionnaire with parental assistance. For dietary behavior, children reported the frequency (days) and intake amount (servings) of fruit, vegetables, meat and meat products, and sugar-sweetened beverages over the previous 7 days. The average daily intake of a single food was determined as [days × (amount in each of those days)]/7. The frequencies of breakfast and intake of high-energy snacks (e.g., chocolates and candies) during the previous week were also reported. For physical activities, children were asked about the average amount of time (minutes) spent per day in sedentary behavior, including sitting and lying, doing homework, watching television, and using computers over the previous 7 days. Three additional questions were asked: “On how many days and for how many hours daily did you perform vigorous-intensity physical activities (e.g., running, basketball, football and swimming) last week? On how many days and for how many hours daily did you perform moderate-intensity physical activities (e.g., table tennis, cycling and dancing.) last week? And on how many days and for how many hours daily did you perform walking last week?”

### 2.4. Statistical Analyses

The participants’ characteristics are described as means (standard deviations, SD) or numbers (%). The student’s *t*-test and the *χ*^2^ test were used to estimate differences continuous and categorical variables, respectively, between subjects delivered by CS and vaginal delivery. Missing data were replaced using the multiple imputation method. To examine the associations between CS and overweight/obesity, crude and adjusted risk ratios (RRs), with 95% confidence intervals (95% CI), were calculated using Poisson regression with robust variance. The minimum set of variables required to enter the adjusted model was identified in a directed acyclic graph using the DAGitty program (version 2.0 alpha, Johannes Textor, Luebeck, Germany) [31,32]. Models were adjusted for birth weight, gestational age, maternal age at childbirth, maternal education level, paternal education level, region, sex, and year of birth (Figure S1). The student’s *t*-test and analysis of covariance (ANCOVA) were used to compare differences in the Z scores between children born via CS and those delivered vaginally (Supplemental Table S3). Similar covariates in Poisson regression were used to adjust the ANCOVA. Subgroup analyses were also conducted according to participants’ age (children 7–12 years vs. adolescents 13–16 years), sex (boys vs. girls), and region (urban vs. rural). A *p* value of less than 0.05 was considered to indicate statistical significance. All analyses were performed using SPSS21.0 (SPSS, Inc., Chicago, IL, USA).

## 3. Results

**Figure 1:**
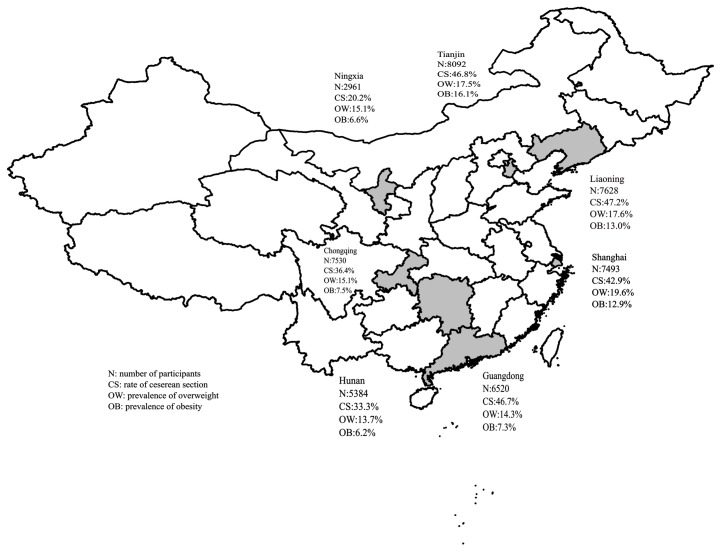
Region-specific distribution of the participants, cesarean section rate and the prevalence of overweight or obesity in China.

**Figure 2:**
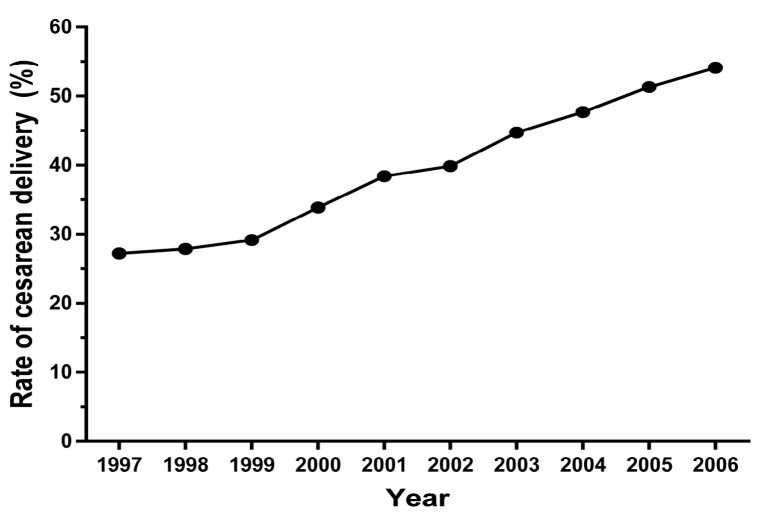
Secular trend in the rate of cesarean section during 1997–2006 in China.

Of the 45,608 students (23,096 boys and 22,512 girls) were enrolled in this national multi-center school-based study, 18,780 (41.2%) were born by CS between 1997 and 2006. The region-specific distributions of the participants, CS rate, and prevalence of overweight/obesity are shown in Figure 1. The overall CS rate increased from 27.2% in 1997 to 54.1% in 2006 (Figure 2).

Among the participants born by CS, 18.5% were overweight and 14.0% were obese. For those delivered vaginally, the estimated rates of overweight and obesity were 15.0% and 8.2%, respectively. 

The maternal and child characteristics of the two exposure groups are shown in Table 1. Mothers who underwent CS had higher education levels and were more likely to have a higher age at pregnancy. CS was also more frequently performed in urban settings, for preterm births, and for offspring with low birth weights. Table S1 presents the dietary and physical activity habits of the groups. Offspring born by CS consumed more meat, fruit, and vegetables per day during the previous week and spent less time in sedentary behavior and physical activities than their peers born via vaginal delivery (*p* < 0.05).

The results of the Poisson regression are shown in Table 2. Crude analysis indicated that children and adolescents born by CS had a 31% (95% CI, 1.26 to 1.37) higher risk of overweight and a 76% (95% CI, 1.66 to 1.86) higher risk of obesity, compared to those delivered vaginally. After adjusting for potential confounders, the increased risk was attenuated but remained. The multivariable adjusted RRs (95% CIs) for overweight and obesity among children and adolescents delivered via CS and those delivered via vaginal birth were 1.21 (1.15 to 1.27) and 1.51 (1.42 to 1.61), respectively. When potential covariates were added to the model, boys born by CS had 13% (95% CI, 1.06 to 1.21) and 45% (95% CI, 1.34 to 1.56) higher risks of overweight and obesity, respectively. Equivalent estimations in girls were 32% (95% CI, 1.22 to 1.42) and 71% (95% CI, 1.51 to 1.93). The increased risks also persisted across subgroups stratified by age and region.

## 4. Discussion

In this nationwide multicenter, school-based study, of a population with a broad age span, we found that the CS rate increased from 27.2% in 1997 to 54.1% in 2006. After controlling for a broad range of major confounding factors, we found that CS was associated with 21% and 51% increases the risks of overweight and obesity, respectively, among offspring. These associations remained similar when the population was stratified by sex, age, and area.

### 4.1. CS Rate

In accordance with the secular trends of CS revealed by previous studies conducted in China [33] and most parts of the world [34], we observed a rapid increase in the CS rate from 1997 to 2006. In addition to clinical indications, a lot of non-clinical reasons may also explain the increasing CS rate in China. First, some women consider CS to be safer and faster, and select this procedure to avoid pain and complications or unwanted outcomes [5]. Second, some women are concerned about genital changes and worry their body figure can’t get back in sharp after vaginal delivery. Third, some Chinese families prefer to select the date of the baby’s delivery on the basis of luck and the baby’s future fate [33]. Additionally, hospitals may encourage scheduled CS delivery as a means of financial profit [35].

However, another important reason may underlie the rapid increase in the CS rate in China. In 2015, the Chinese government repealed the one-child policy of nearly 40 years’ duration and implemented a universal two-child policy [36]. This policy will encourage many families to have a second child. As with our study, however, many previous studies, have indicated that pregnant women of advanced maternal age prefer a CS delivery [37,38] and that expectant mothers in their second pregnancies are more likely to choose a CS delivery if they had a previous CS [38]. Therefore, the CS rate may further increase sharply in China.

Although clinically indicated CS can effectively reduce maternal and perinatal mortality and morbidity rates, no evidence has demonstrated benefits from non-essential CS [34]. Moreover, CS has been recognized as a risk factor for short-term lung function impairment, hypoglycemia, reduced breast feeding initiation [10,39], and long-term detrimental effects on immune-related conditions, such as asthma [7], respiratory morbidity [8], and type 1 diabetes [7]. In addition, as with any surgery, CS may also impose short- and long-term negative effects on the health of the woman and her future pregnancies [40,41]. Therefore, the Chinese government should develop specific policies and measures to effectively control the rate of CS.

### 4.2. The Association between CS and Offspring Overweight and Obesity

The observed direct association between CS and overweight and obesity in this study is consistent with two previous meta-analyses [17,18]. Both studies indicated that children delivered by CS had a moderately increased risk of obesity (33% and 34%) when compared with vaginally delivered children. In contrast, a recent bias-adjusted meta-analysis reported a null effect of CS on childhood overweight and obesity and found that residual confounding and publication bias might have contributed to the observed effects of CS on offspring overweight and obesity in the previous two meta-analyses [19].

The results of a recent large cohort study in the United States with a series of sensitivity analyses indicated that offspring born by CS exhibited a 15% (decrease from 30% in the crude model) increased risk of obesity after adjusting for maternal age at delivery, race, region, year of birth, pre-pregnancy BMI, maternal height, gestational diabetes, preeclampsia, pregnancy-induced hypertension, gestational age at delivery, birth weight, pre-pregnancy smoking, previous CS, and offspring sex [20]. In a Dutch birth cohort study of 2641 children, Pluymen et al. reported that the OR (95% CI) for the association of CS with overweight decreased from 1.69 (1.35 to 2.13) to 1.52 (1.18 to 1.96) after excluding life style, behavior, and other potential confounders [42]. In agreement with those studies, CS also independently predicted the weight status of offspring in our data, which indicates that further adjustment for additional relevant variables might reduce the calculated effect, but the effect of CS is unlikely to be nullified.

Our stratified estimates indicated that CS correlated significantly with adiposity in both children and adolescents. This is in line with findings from two longitudinal studies that used generalized linear mixed models, which found an increased risk of overweight that persisted throughout childhood in children delivered by CS [42,43]. In accordance with the subgroup meta-analysis regarding gender-specific estimates by Li et al. [18], we also observed that sex did not modify the shape of the association between a higher BMI and CS. When stratified by region, similar CS-obesity associations were observed in subjects from urban and rural areas. This finding was supported by the meta-analysis of Kuhleet et al., which showed that effect estimates did not vary by country income [17].

### 4.3. Potential Mechanisms

Although the mechanism underlying the association of CS with adiposity requires further exploration, a growing body of research has demonstrated that intestinal flora may play an important role [44,45]. Because infants delivered by CS are not exposed to maternal vaginal and intestinal flora, their gut microbiota composition and colonization patterns differ from those who are delivered vaginally [46]. Compared with those born vaginally, newborns delivered by CS have fewer populations of *Bifidobacteria* and *Bacteroides* species in their gastrointestinal tracts, and the colonizing microbiota is less bacterially diverse [45]. This aberrant gut microbiota composition and colonization may be associated with the capacity for energy harvesting and the risks of overweight and obesity in later life [47]. Additionally, epidemiological evidence suggests that CS correlates with a lower umbilical leptin concentration [48] and a reduced rate of early breastfeeding [49], both of which were reported to be associated with an increased risk of later obesity [50,51].

### 4.4. Strengths and Limitations

Although this topic has been explored widely in different populations, the findings of relevant individual studies remain inconsistent. Our findings extend and refine the evidence in this area due to several strengths. The national multicenter school-based design guaranteed that our study is widely representative. The large sample size and broad age range allowed us to achieve sufficient power to examine the association between CS and the risk of overweight and obesity in children and adolescents. The availability of key parent and offspring information, especially lifestyle factors such as dietary, sedentary behaviors, and physical activity, allowed us to address residual confounding, estimate adjusted effect sizes, and examine the robustness of associations by subgroup analyses. In addition, the weights and heights of offspring were measured by trained health staff using calibrated instruments, and thus misclassification was very unlikely.

However, the study also had some limitations. First, despite adjusting for a large number of confounders related to maternal, child, and socioeconomic factors, the possibility of residual confounding could not be eliminated, especially involving information indicating for CS, antibiotic use during pregnancy, pre-pregnancy maternal BMI and gestational weight gain. However, in a 15-year follow-up study, a significant correlation between pre-pregnancy weight and subsequent weight (*r* = 0.74) was suggested [52]. Thus, the adjustment for current maternal BMI partially addresses the possible confounding effect of pre-pregnancy BMI. Second, our study was cross-sectional and collected prenatal and postnatal information retrospectively. However, any bias is likely to be non-differential within the context of a general health survey. Several studies have shown that the maternal retrospective recall of gestational age, birth weight, and breastfeeding history is both reproducible and valid for clinical and epidemiological research [53]. Third, lifestyle information was not collected prospectively. Some obese children might have already changed their eating and physical activity habits before enrollment. Therefore, the CS—obesity relationship was somewhat overestimated due to prevalence-incidence bias. Fourth, our data lacked the indications for CS, and specifically elective CS may correlate with childhood adiposity. However, findings from 9103 Chinese preschool children showed a significant increased risk of overweight and obesity among children delivered by elective and non-elective CS [21]. Fifth, the missing covariate data frequencies ranged from 2.5% to 13.0% in our study. However, analyses conducted after the multiple imputation of missing values yielded findings similar to the complete case analysis (data not shown), and therefore the effects of the missing values on the results were minimal. Sixth, we lacked data on the participants’ gut microbiota or other potential mediators that would allow further exploration of the underlying mechanisms. Finally, we could not distinguish between elective and emergency CS. In a birth cohort study of 10,219 children, Blustein et al. reported that the effect of CS on childhood obesity was more robust for elective than emergency CS [43]. Further studies are needed to assess this difference in the Chinese population.

### 4.5. Clinical Implication

Based on this national multicenter school-based study, we suggest that CS represents an early-life risk factor for later childhood and adolescent obesity. Strategies are urgently needed to effectively control the rate of CS given the implications of the universal two-child policy in China. Clinicians and patients should weigh the potential outcomes when considering cesarean birth in the absence of a clear medical or obstetric indication. Furthermore, children who born via CS may require additional monitoring.

## 5. Conclusions

This study observed a secular trend of an increasing CS rate from 1997 to 2006. Children and adolescents born by CS tended to have higher risks of becoming overweight and obese compared to those born by vaginal delivery.

## Figures and Tables

**Table 1:** Characteristics of children and adolescents by different delivery mode.

Characteristics	Mean (SD)/*n*(%)			*p*
	Total (*n* = 45,608)	Cesarean Delivery (*n* = 18,780)	Vaginal Delivery (*n* = 26,828)	
Sex				<0.001
Boys	23,096(50.6)	9881(52.6)	13,215(49.3)	
Girls	22,512(49.4)	8899(47.4)	13,613(50.7)	
Age (year)	10.9(2.9)	10.2(2.7)	11.4(2.9)	<0.001
Height (cm)	146.9(15.6)	144.3(15.5)	148.7(15.5)	<0.001
Weight (kg)	41.7(15.0)	40.3(15.3)	42.6(14.8)	<0.001
BMI (kg/m^2^)	18.7(3.8)	18.7(4.0)	18.7(3.7)	0.527
BMI-Zscore	0.24(1.29)	0.41(1.34)	0.12(1.24)	<0.001
Year of birth				<0.001
1997–2001	30,361(66.6)	14,341(76.4)	16,020(59.7)	
2002–2006	15,247(33.4)	4439(23.6)	10,808(40.3)	
Overweight	7481(16.4)	3468(18.5)	4013(15.0)	<0.001
Obesity	4831(10.6)	2620(14.0)	2211(8.2)	<0.001
Region				<0.001
Urban	28,396(62.3)	13,031(69.4)	15,365(57.3)	
Rural	17,212(37.7)	5749(30.6)	11,463(42.7)	
Gestational age (weeks)	39.6(1.3)	39.4(1.4)	39.7(1.2)	<0.001
<37	1175(2.5)	624(3.3)	551(2.1)	
37–42	41,983(92.1)	17,156(91.4)	24,827(92.5)	
≥42	995(2.2)	420(2.2)	575(2.1)	
Missing	1455(3.2)	580(3.1)	875(3.3)	
Birth weight (grams)	3322.4(503.6)	3352.9(510.0)	3300.7(497.8)	<0.001
<2500	1459(3.2)	850(3.2)	609(3.2)	
2500–4000	37,335(81.9)	22,084(82.3)	15,251(81.2)	
≥4000	4308(9.4)	2255(8.4)	2053(10.9)	
Missing	2506(5.5)	1639(6.1)	867(4.6)	
Breast feeding (months)	8.4(6.0)	7.7(6.0)	8.9(6.0)	<0.001
<6	12,074(26.5)	5969(31.8)	6105(22.8)	
≥6	27,099(59.4)	10,496(55.9)	16,603(61.9)	
Missing	6435(14.1)	2315(12.3)	4120(15.3)	
Parental characteristics				
Maternal age at birth (year)	26.0(4.3)	26.5(4.3)	25.6(4.3)	<0.001
Mather’s BMI (kg/m^2^)	22.2(3.1)	22.4(3.1)	22.1(3.0)	<0.001
Father’s BMI (kg/m^2^)	24.2(3.3)	24.3(3.3)	24.0(3.3)	<0.001
Maternal education (years)				<0.001
≤6	4409(9.7)	984(5.2)	3425(12.8)	
7–12	28,434(62.3)	10,883(58.0)	17,551(65.4)	
≥13	11,563(25.4)	6551(34.9)	5012(18.7)	
Missing	1202(2.6)	362(1.9)	840(3.1)	
Paternal education (years)				<0.001
≤6	3208(7.0)	784(4.2)	2424(9.0)	
7–12	28,628(62.8)	10,722(57.1)	17,906(66.7)	
≥13	12,651(27.7)	6939(37.0)	5712(21.3)	
Missing	1121(2.5)	335(1.8)	786(2.9)	

**Table 2:** Crude and multivariable adjusted risk ratios for overweight/obesity in offspring associated with cesarean vs. vaginal delivery.

Group	Overweight (RR (95% CI))					Obesity (RR (95% CI))				
	Crude	*p*	Adjusted	*p*	*p*a	Crude	*p*	Adjusted	*p*	*p*a
Total	1.31 (1.26, 1.37)	<0.001	1.21 (1.15, 1.27)	<0.001	---	1.76 (1.66, 1.86)	<0.001	1.51 (1.42, 1.61)	<0.001	---
Stratified analysis by sex										
Boys	1.23 (1.16, 1.31)	<0.001	1.13 (1.06, 1.21)	<0.001	<0.001	1.64 (1.53, 1.75)	<0.001	1.45 (1.34, 1.56)	<0.001	<0.001
Girls	1.40 (1.31, 1.50)	<0.001	1.32 (1.22, 1.42)	<0.001	<0.001	1.86 (1.66, 2.08)	<0.001	1.71 (1.51, 1.93)	<0.001	<0.001
Stratified analysis by age										
Children	1.21 (1.14, 1.27)	<0.001	1.18 (1.12, 1.26)	<0.001	<0.001	1.55 (1.45, 1.65)	<0.001	1.49 (1.39, 1.60)	<0.001	<0.001
Adolescents	1.42 (1.30, 1.55)	<0.001	1.29 (1.16, 1.43)	<0.001	<0.001	1.86 (1.64, 2.10)	<0.001	1.57 (1.36, 1.80)	<0.001	<0.001
Stratified analysis by region										
Urban	1.37 (1.29, 1.45)	<0.001	1.24 (1.16, 1.32)	<0.001	0.001	1.94 (1.80, 2.09)	<0.001	1.58 (1.46, 1.72)	<0.001	<0.001
Rural	1.23 (1.14, 1.33)	<0.001	1.15 (1.06, 1.25)	<0.001	0.002	1.53 (1.39, 1.68)	<0.001	1.39 (1.26, 1.54)	<0.001	<0.001

*p* Values refer to Wald’s test. *p*a: *p* value for interaction between stratified variables and delivery mode. Analyses were adjusted for birth weight, gestational age, maternal age at childbirth, maternal education level, paternal education level, region, sex, and year of birth. The sex-subgroup analysis was adjusted for all covariates except for sex; the area-subgroup analysis was adjusted for all covariates except for area.

## Supplementary Materials

The following are available online at www.mdpi.com/1660-4601/14/12/1609/s1, Figure S1: Directed acyclic graph (DAG) illustrating relationship of delivery mode and overweight/obesity and confounders, Table S1: Lifestyle of children and adolescents by different mode of delivery; Table S2: Crude and multivariable adjusted risk ratios for obesity in offspring associated with cesarean vs. vaginal delivery; Table S3: Differences of BMI z-scores between cesarean and vaginal delivery (means ± SE).
