# Supplementary material for: Association between Cesarean Section and Weight Status in Chinese Children and Adolescents: A National Survey
Source: Int J Environ Res Public Health. 2017 Dec 20;14(12):1609. doi: 10.3390/ijerph14121609 (PMC5751025; doi:10.3390/ijerph14121609)
Supplement: Supplementary file 1 [file ijerph-14-01609-s001.pdf]

Supplementary Material

Association between Cesarean Section and Weight Status in Chinese Children and Adolescents: A National Survey

Jingjing Liang [1,†], Zheqing Zhang [2,†], Wenhan Yang [1], Meixia Dai [1], Lizi Lin [1], Yajun Chen [1], Jun Ma [3] and Jin Jing [1,*]

Table S1. Lifestyle of children and adolescents by different mode of delivery.

Lifestyle	N	Total	Cesarean delivery	Vaginal delivery	*p*
Dietary behavior					
Fruit (servings/d)[a]	43374	1.31(1.10)	1.36(1.13)	1.27(1.08)	<0.001
Vegetable (servings/d)[a]	43432	1.83(1.46)	1.88(1.48)	1.81(1.44)	<0.001
Sugar-sweetened beverages (cups/d)[b]	42584	0.40(0.74)	0.36(0.68)	0.42(0.78)	<0.001
Meat (servings/d)[c]	43291	1.18(1.22)	1.23(1.25)	1.15(1.20)	<0.001
breakfast (day/week)	43907	6.49(1.42)	6.59(1.29)	6.42(1.50)	<0.001
snacks (day/week)	43578	2.01(1.95)	1.93(1.90)	2.06(1.99)	<0.001
Sedentary behavior (minute/d)					
Sitting and lying	39609	336.7(221.3)	332.8(216.9)	339.4(224.4)	0.004
Doing homework	42716	116.2(73.4)	112.2(72.2)	119.1(74.0)	<0.001
Watching television	41431	55.4(62.7)	52.2(57.3)	57.6(66.2)	<0.001
Using computer	39525	41.8(66.2)	37.3(61.3)	44.9(69.3)	<0.001
Physical activity (minute/d)					
Vigorous intensity physical activity	41274	28.7(44.7)	27.9(42.8)	29.3(46.0)	0.002
Moderate intensity physical activity	40882	28.4(44.2)	27.3(42.4)	29.3(45.4)	<0.001
Walking	41167	45.7(67.02)	43.2(64.4)	47.5(68.7)	<0.001

[a]: A serving of fruit or vegetable is equivalent to 100 g ; [b]: A cup is equivalent to 250 mL; [c]: A serving of meat products is equivalent to 75 g

Table S2. Crude and multivariable adjusted risk ratios for obesity in offspring associated with cesarean vs. vaginal delivery*

Group	Overweight (RR (95%CI))				Obesity (RR (95%CI))			
	Crude	P	Adjusted	P	Crude	P	Adjusted	P
Total	1.30 (1.23, 1.38)	<0.001	1.20 (1.13, 1.28)	<0.001	1.56 (1.49, 1.64)	<0.001	1.37 (1.30, 1.45)	<0.001
Stratified analysis by sex								
Boys	1.22 (1.13, 1.32)	<0.001	1.13 (1.04, 1.24)	0.004	1.46 (1.38, 1.54)	<0.001	1.31 (1.23, 1.39)	<0.001
Girls	1.39 (1.28, 1.51)	<0.001	1.29 (1.18, 1.42)	<0.001	1.65 (1.51, 1.79)	<0.001	1.54 (1.40, 1.69)	<0.001
Stratified analysis by age								
Children	1.20 (1.12, 1.28)	<0.001	1.17 (1.09, 1.26)	<0.001	1.46 (1.38, 1.54)	<0.001	1.31 (1.23, 1.39)	<0.001
Adolescents	1.40 (1.25, 1.56)	<0.001	1.29 (1.14, 1.47)	<0.001	1.65 (1.51, 1.79)	<0.001	1.54 (1.40, 1.69)	<0.001
Stratified analysis by region								
Urban	1.36 (1.27, 1.46)	<0.001	1.23 (1.13, 1.33)	<0.001	1.69 (1.59, 1.79)	<0.001	1.42 (1.33, 1.52)	<0.001
Rural	1.23 (1.12, 1.35)	<0.001	1.16 (1.05, 1.28)	0.005	1.39 (1.28, 1.50)	<0.001	1.28 (1.17, 1.39)	<0.001

* Overweight and obesity are defined according to the "BMI percentile" cutoff values proposed by the WHO. P values refer to Wald's test. Analyses were adjusted for birth weight, gestational age, maternal age at childbirth, maternal education level, paternal education level, region, sex, and year of birth. The sex-subgroup analysis was adjusted for all covariates except for sex; the area-subgroup analysis was adjusted for all covariates except for area.

Table S3. Differences of BMI z-scores between cesarean and vaginal delivery (means ± SE)

Group	Cesarean delivery	Vaginal delivery	P-crude	Cesarean delivery	Vaginal delivery	P-adjusted
Total	0.411 ± 0.009	0.118 ± 0.008	<0.001	0.384 ± 0.010	0.157 ± 0.009	<0.001
Stratified analysis by sex						
Boys	0.630 ± 0.014	0.293 ± 0.012	<0.001	0.598 ± 0.015	0.352 ± 0.013	<0.001
Girls	0.168 ± 0.012	-0.053 ± 0.010	<0.001	0.169 ± 0.013	-0.039 ± 0.011	<0.001
Stratified analysis by age						
Children	0.467 ± 0.011	0.223 ± 0.010	<0.001	0.456 ± 0.012	0.232 ± 0.011	<0.001
Adolescents	0.232 ± 0.018	-0.038 ± 0.011	<0.001	0.227 ± 0.020	-0.002 ± 0.013	<0.001
Stratified analysis by region						
Urban	0.412 ± 0.011	0.077 ± 0.010	<0.001	0.383 ± 0.012	0.134 ± 0.011	<0.001
Rural	0.409 ± 0.017	0.172 ± 0.012	<0.001	0.375 ± 0.018	0.193 ± 0.013	<0.001

P values refer to ANCOVA test. Analyses were adjusted for birth weight, gestational age, maternal age at childbirth, maternal education level, paternal education level, region, sex, and year of birth. The sex-subgroup analysis was adjusted for all covariates except for sex; the area-subgroup analysis was adjusted for all covariates except for area.

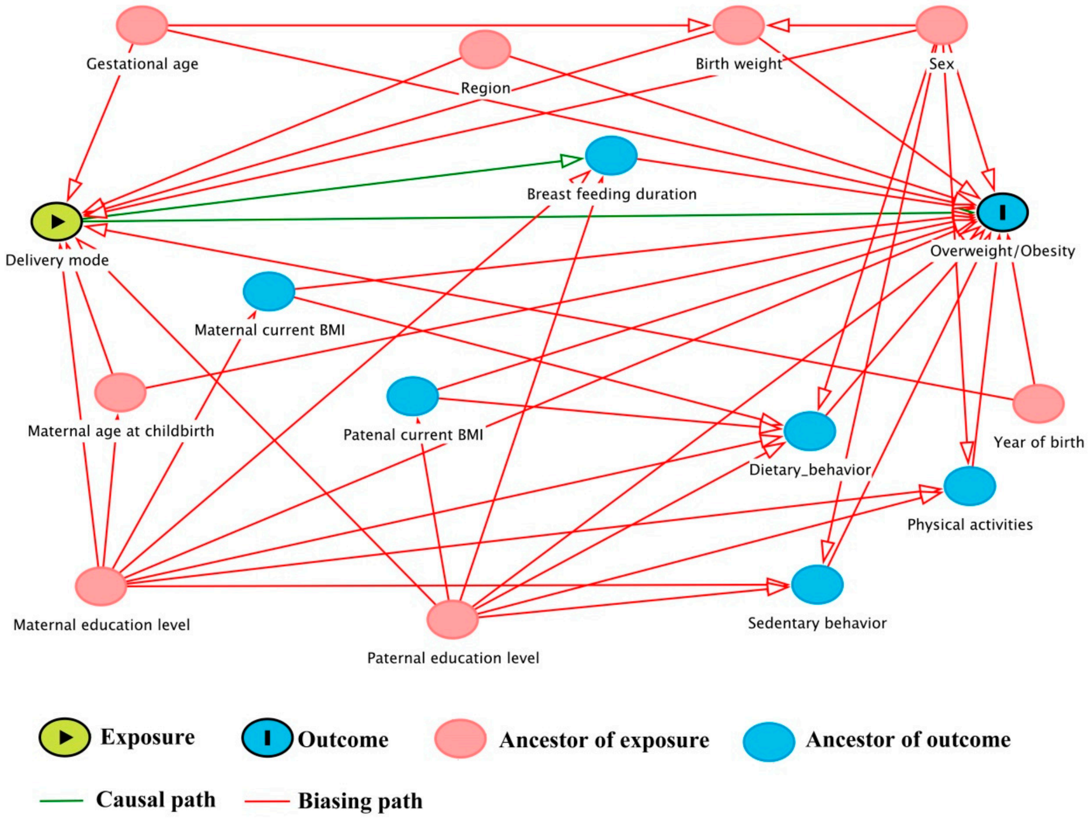

Figure S1. Directed acyclic graph (DAG) illustrating relationship of delivery mode and overweight/obesity and confounders
